# Assessment of the Present and Future Offshore Wind Power Potential: A Case Study in a Target Territory of the Baltic Sea Near the Latvian Coast

**DOI:** 10.1155/2013/126428

**Published:** 2013-07-29

**Authors:** Lita Lizuma, Zanita Avotniece, Sergejs Rupainis, Artis Teilans

**Affiliations:** ^1^Institute of Physical Research and Biomechanics, Artilerijas 40, Riga 1090, Latvia; ^2^Faculty of Geography and Earth Sciences, University of Latvia, Alberta 10, Riga 1010, Latvia; ^3^Rezekne Higher Education Institution, Atbrivosanas aleja 90, Rezekne 4601, Latvia

## Abstract

Offshore wind energy development promises to be a significant domestic renewable energy source in Latvia. The reliable prediction of present and future wind resources at offshore sites is crucial for planning and selecting the location for wind farms. The overall goal of this paper is the assessment of offshore wind power potential in a target territory of the Baltic Sea near the Latvian coast as well as the identification of a trend in the future wind energy potential for the study territory. The regional climate model CLM and High Resolution Limited Area Model (Hirlam) simulations were used to obtain the wind climatology data for the study area. The results indicated that offshore wind energy is promising for expanding the national electricity generation and will continue to be a stable resource for electricity generation in the region over the 21st century.

## 1. Introduction 

The exploitation of renewable energy sources can help the European Union meet many of its environmental and energy policy goals, including its obligation to reduce greenhouse gases under the Kyoto Protocol and the aim of securing its energy supply [1]. 

The current situation regarding wind energy production in Latvia is unsatisfactory. According to the data from Latvenergo AS, approximately 1535 MW (76%) of the total electricity production in Latvia, is generated by hydroelectric power plants, while about 474 MW (23%) is generated at thermal power plants and from fossil fuels, but only around one percent is generated by wind power. Offshore wind energy development promises to be a significant domestic renewable energy source for Latvia.

The first stage of exploiting the wind energy is the evaluation of wind resources at wind farm sites which means a site-specific evaluation of wind climatology and vertical profiles of wind, as well as the assessment of historical and potential future changes in the wind climate. Suitable long-term wind observation data are needed for this purpose. Reliable prediction of the wind resources as well as site conditions at offshore sites is crucial for project planning and selecting suitable locations. There is a lack of such data sets in the focus area of this study. In situ measurements of wind and environmental conditions are available only for the coastal areas. 

 There are different ways of estimating the wind resources at a site: interviews of people with local knowledge to identify areas with high and/or low wind speed, measurements only, the measure-correlate-predict method, using global databases, wind atlas methodology, site data-base modelling, and mesoscale and microscale modelling [2, 3]. Recently offshore wind energy mapping has benefited from the advantages provided by the remote sensing data [4–6].

The infrastructure of large offshore installation is typically more expensive than onshore counterparts, relying on somewhat even longer depreciation time. For understanding how the global climate change might influence the magnitude of wind resources as well as the operation and maintenance conditions of wind turbines the General Circulation (or Global Climate) Models (GCMs) and regional climate downscaling (i.e., use of GCM outputs in higher spatial and temporal resolution models) are the primary tools for developing such projections [3]. The method of using a Regional Climate Models (RCMs) to assess the impact of climate change on the wind energy resource has been used before [7, 8].

The overall goal of this paper is the assessment of offshore wind power potential in a target territory of the Baltic Sea near the Latvian coast as well as the identification of a trend (if any) in the future wind energy potential for the study territory. 

## 2. Datasets and Calculation Methods

**Figure 1:**
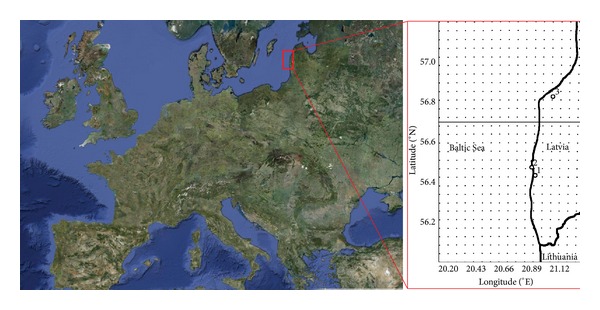
The study area with sites of In situ observation stations and grid point locations. 1: weather station Liepaja 1; 2: weather station Liepaja 2; 3: weather station Pavilosta.

The assessment of the wind climate was performed for the study region domain extending from 56.03N 20.2E to 57.22N 21.33E (Figure 1). The previous studies have shown that this territory is with a high wind energy generation potential [1]. Several wind turbines are planned to be mounted here in the near future. 

### 2.1. Models Datasets

In this stage of investigation we focus on the assessment of the long-term present and future conditions. The method of regional climate modelling was used. The regional climate model and high resolution operational model simulations were used to obtain the meteorological and climatological data for the study area. 

The RCM simulations used in this research were obtained from ENSEMBLES EU FP6 project (http://www.ensembles-eu.org/) [9–11]. The RCM simulations from ENSEMBLES project have been extensively evaluated [12, 13] and ETHZ-CLM_SCN_HadCM3Q0_25 km (CLM) were chosen for obtaining the climate data. CLM is the climate version of the “Local Model,” the operational weather forecast model of the Consortium for Small scale Modelling (COSMO). The consortium was formed by a number of meteorological services including the German Meteorological Service and Meteo Swiss with the goal to “develop, improve, and maintain a nonhydrostatic limited-area atmospheric model, to be used both for operational and for research applications” (http://www.cosmo-model.org/). The results used in this study are from two runs of CLM model, one with the boundary conditions from ERA-40 for the control period 1981–2010 and one with a single set of lateral boundary conditions from ECHAM5 for two future periods 2021–2050 and 2071–2100 applied for the IPCC scenario A1B. The A1B scenario equals to the future world of very rapid economic growth, global population that peaks in the mid-century and declines thereafter, and the rapid introduction of new and more efficient technologies with balance across all sources [14]. The sensitivity of the projections to differences between emissions scenarios was found to be small prior to about 2040, but it increases substantially in the second half of the 21st century [15]. As the emission scenario uncertainty is still relatively small for the period preceding 2050, we focused on climate change under the A1B scenario. 

The meteorological data were obtained also from the Baltic Operational Oceanography System forecast model (DMI-Hirlam) and the EU Project MyOcean FiMAr information system [16]. The data from Hirlam model covers the period 2005–2010. 

The modelled data of wind speed, wind direction, air temperature, and atmospheric pressure were used. All meteorological parameters are presented at 10-m, 80 m, 90 m, and 100 m height.

The atmospheric components of CLM were interpolated using Kriging interpolation method from grid cells with horizontal 25 km × 25 km to resolution of 5 × 5 km. Grid cells points are shown in Figure 1. For data comparison purpose the same grid points were used for Hirlam model data. The details of the model data are given in Table 1.

### 2.2. In Situ Observation Data

Direct measurements from meteorological stations within the study area were obtained from three costal stations (Figure 1). All the stations measured winds at 10 m height. The meteorological stations Liepaja 1 and Pavilosta covered 30 years of observations. Meteorological station Liepaja 2 is located very close to the sea coast and covered 7 years of observations. The details of the in situ observation data are given in Table 2. It should be noted that taking into account the local site conditions the wind measurement data are representative for the actual measurement site. When comparing the model output with the observed station data, the model data were interpolated onto the latitude-longitude station location. The estimation and elimination of local effect associated with site conditions (obstacles and roughness length) as well as complexity of coastal winds were carried out. The long-terms average monthly and annual wind speed data of stations Liepaja 1 and Pavilosta were adjusted to roughness length 0.03. 

### 2.3. Meteorological Satellite Data

The data from CLM and Hirlam models were compared with data from meteorological satellites. The wind speed data at 10 m were obtained from EUMETSAT established Satellite Application Facility (SAF) on Climate Monitoring (CM SAF, http://www.cmsaf.eu/) dataset named the “Hamburg Ocean-Atmosphere Parameters and Fluxes from Satellite Data” (HOAPS) [17]. Satellite data on the wind speed at 10 m height cover a time period 1998–2005; however, the data are available only at a distance from the coast, depending on the resolution with which the data were obtained. In some places, this may imply that observations are available only over deep water. Even in these areas, wind statistics from satellites can provide the basic starting point to decide where to focus attention for wind energy purposes. In this study available satellite observation data points which were the closest to the territory of interest were used for the model data evaluation. 

### 2.4. Calculation Methods

The mean wind speed and the mean cubed wind speed were calculated at each grid point per month, per season, and per day. The mean absolute difference metric (MAD) and the root mean square difference metric (RMSD) were used to compare the CLM and Hirlam simulations with the in situ and satellite observation data.

 The “projected percentage change” metric was used to give a measure of expected climate change by comparing the future climate projections with the control simulation. It is defined as follows:

(1) $$\begin{array}{c} D_{i}=100*\frac{\left(F_{i}-P_{i}\right)}{P_{i}}, \end{array}$$

where *i* is the grid point, and *P*_*i*_ and *F*_*i*_ are the wind data for the past and future model runs, respectively.

The wind power density (WPD) was calculated as follows:

(2) $$\begin{array}{c} \text{WPD}\left(\text{W}/{\text{m}}^{2}\right)=0.5*\frac{1}{n}*\sum \left(\rho j*{Vj}^{3}\right), \end{array}$$

where *V* is the wind speed reading and *ρ* is air density and *n* is the number of readings. 

Since the air temperature and pressure data are available, the air density was calculated as follows:

(3) $$\begin{array}{c} \rho \;=\frac{P}{RT}\;\left(\text{kg}/{\text{m}}^{3}\right), \end{array}$$

where *P* = air pressure (in units of Pascals), *R* = the specific gas constant (287 J kg^−1^ Kelvin^−1^), and *T* = air temperature in degrees Kelvin (deg. C + 273).

## 3. Results and Discussion

### 3.1. Comparison of Model and In Situ Data

Evaluation of wind speed and direction, air temperature, and atmospheric pressure data from models and in situ data was performed by comparing the mean annual, seasonal, and daily data from three meteorological in situ observation stations (Figure 1) and CLM and Hirlam data at 10 m height. 

A good agreement was found between the long-term modelled and observed wind speed data sets in terms of long-term month-to-month variability. The CLM overestimates the long-term annual wind speed by 0.1 m/s (Table 3(a)). On seasonal base the MAD does not exceed 0.3°C. The simulated wind shows generally the same directional pattern as the measurements. The bias of air temperature for the long-term seasonal means does not exceed 0.5°C (Table 3(a)). The observed daily data have a higher correlation coefficient (0.84–0.93 for wind speed; 0.95–0.99 for air temperature; and 0.98-0.99 for air pressure) with the Hirlam data (Table 3(b)) than with CLM data (0.55–0.76 for wind speed; 0.65–0.77 for air temperature; and 0.70–0.89 for air pressure). Comparing the Hirlam simulations and Liepaja 1 and Liepaja 2 in situ daily observations (period 2005–2010) the difference statistics are MAD = 0.3 m/s and 0.6 m/s; RMSD = 1.0 m/s and 0.8 m/s. 

It was found that in general the long-term average wind climate predictions of CLM and Hirlam are in good agreement with the in situ measurements. Hirlam model also shows very good agreement with the observation data on daily basis.

### 3.2. Comparison of Global Model and Satellite Data

**Figure 2:**
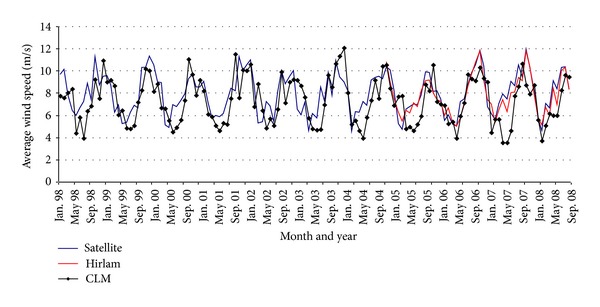
Time series of monthly average wind speed calculated from CLM, Hirlam, and HOAPS data at grid point 56.03N 20.12E.

Monthly average wind speed at 10 m derived from the HOAPS data base was compared with simulation results of the CLM and Hirlam models. During the comparison it was found that a mean absolute difference of 0.3 m/s to 1.0 m/s is evident for Hirlam and CLM data sets, respectively. The bias is mostly positive, and the satellite winds tend to be larger than those of the atmospheric models.  Figure 2 illustrates a high agreement between the monthly average wind speed at 10 m height with the correlation coefficients 0.95 (Hirlam) and 0.66 (CLM). The fluctuations of model data around the satellite readings suggest that the small disagreement is due to the differences in temporal resolutions between these data sets. In Figure 2 the time series of monthly average wind speed from modelled and satellite data are illustrated in the proximity of (56.03N 20.12E), located on our grid. 

In general the results of this study have shown that both model data are complementary to observations at meteorological stations and satellite readings and can be used for meaningful mapping and assessment of the long-term wind climate and wind power resources for the target offshore area. The Hirlam model has shown a better agreement with the observation data and could be used for an accurate wind regime and wind resources assessment. CLM model appears to be a valuable data source upon which one could base long-term monthly or yearly wind resources estimates and forecasts. 

### 3.3. Results of Present Wind Climate Assessment

The annual wind power potential has a high economical impact for wind parks. The annual power production varies with the wind speed, air temperature and pressure, and from that by the climatology of these elements. The standard procedure to determine the climatology of wind and other meteorological parameters has been to look at 30-year averages (WMO-sanctioned norm), typically the period 1961–1990. In this work we chose to update the 30-year period to a more recent one, encompassing the later part of our data set, 1981–2010. Consequently, the last decade with relative strong anthropogenic impact on the climate was included. However our data from Hirlam model which has shown the better agreement with observation data covers only a 6-year period 2005–2010. In order to assess the differences in the wind climatology between two time periods 1981–2010 and 2005–2010, we evaluated the CLM data for these two time periods. Test shifting the reference period to 2005–2010 showed negligible differences in the results. The average annual wind speed differs from 0.0 to 0.2 m/s and calculated annual wind power density differs from 0 to 3%, respectively, in 492 grid points. 

**Figure 3:**
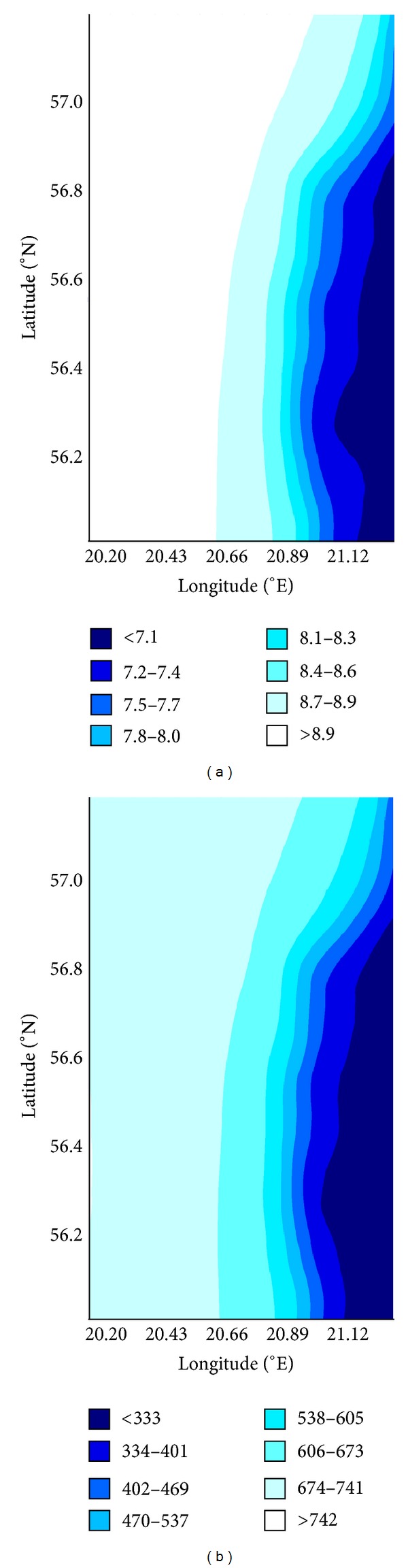
(a) Annual average wind speed at 100 m height for the period 2004–2010 based on the Hirlam model data. (b) Average wind power density at 100 m.

Figure 3 shows the average annual wind speed at 100 m for the time period 2005–2010 and annual average wind power density. A clear gradient in both variables can be observed from the open sea to coastal areas.

The created maps are based on the atmospheric model data that provide a better estimate of the offshore wind resources than the previously available evaluation using the in situ observation data from the coastal stations. In terrestrial territories the wind speeds vary dramatically in the horizontal direction a few kilometres or even hundreds of meters over the sea; however, there are strong local effects near the coasts, but at the open sea much larger areas have fairly uniform wind speeds. Wide areas of similar wind speed can be seen for the region covered in maps. For the most of the sea territory the annual mean wind speed is 6–7.5 m/s at 10 m and 7.5–8.5 m/s at 100 m (Figure 3). The monthly average wind speed in the sea territory varies from 7.5–9.5 m/s in winter to 4-5 m/s in summer at 10 m height and from 9–11 m/s in winter to 6–8 m/s in summer at 100 m height. By contrast, the terrestrial sites have substantially lower wind speed and power density. For all locations and all heights the winter averages are about 1.8 : 1 greater than those of summer. The wind power density increases rapidly by crossing the coast headed seawards. Open sea sites result in about 2.5 to 1.5 greater wind power density than terrestrial locations. The wind power density in all teritory is typically 300–700 W/m^2^. Offshore, the resources are predicted to be more than 500 W/m^2^. 

**Figure 4:**
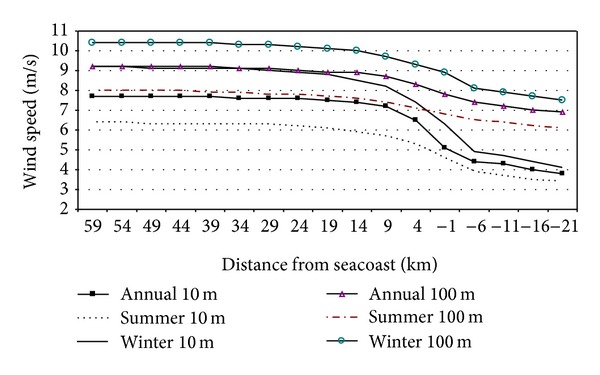
Mean annual and seasonal (winter-DJF, summer-JJA) wind speed at 10 and 100 m height (2004–2010). The location of the transect is indicated in Figure 1.

A horizontal transect shown in Figure 4 visualizes the variations in mean wind speed from the coastline to the open part of the sea and illustrates the seasonal variability of the wind speed. The figure shows a significant increase in the wind speed up to 10 km from the coastline to the open sea areas and a slight decrease with the increasing distance from the coast to inland. There are very little changes in the wind speed at the distances from 20 to 60 km from the coast to the sea. 

Table 4 gives the summary statistics of wind speed characteristics at 100 m for 4 sites with different distance from the seacoast. Site I is located in a coastal area very close to the sea. Another three (II–IV) sites are located in offshore sites and present the locations for offshore wind farms in the near future. 

Variations in the annual wind speed and related wind energy density are also of great importance for the wind farm development. According to 30-year CLM datasets, the range of annual wind speed indicates that the annual wind speed for any given year within a 30-year period (1981–2010) may be over 3% higher or lower than the mean wind speed during the period. Higher degree of interannual variability was found for monthly average wind speed: up to 20% in spring (April) and autumn (September) and up to 10% in summer. 

For calm to weak winds, the wind turbines are at halt. The wind speed at which the turbine first starts to rotate and to generate a power is typically between 3 and 4 m/s. To understand consistency of electrical output, we examined the persistence of wind speeds exceeding 4 m at different locations and different heights. Annual mean percent active (speed > 4 m/s) daily values at a 100 m height range from 92 to 95% for all of the offshore sites, while the terrestrial sites at 100 m height are active 88 to 90% of the time.  Table 5 illustrates the annual mean percentage of days with active wind speed for four sites at different heights. The results show that for the offshore territories there are no significant differences in the occurrence of active wind speed at the heights 80–100 m. The occurrence of active wind speed increases noticeably between 10 m and 80 m height particularly for the terrestrial site. 

In general, the results of wind speed mapping and wind power potential assessment indicated that offshore wind resources in the territory are promising to expand national electricity generation. 

### 3.4. Long-Term Changes of Wind Climate

Like many other renewable technologies wind energy is also susceptible to climate change. The principal and most direct mechanism by which global climate change may impact the wind energy industry is by changing the geographical distribution and/or the inter- and intra-annual variability of the wind resources [18]. The analysis of the changes in long-term historical wind speed data was beyond the scope of this study. However it should be mentioned that the trend analysis of the average wind speed and maximum wind gusts discloses the decreasing trends in coastal observation stations Liepaja 1 and Pavilosta. In general the results of the changes in the wind climate from two costal weather stations were compared with the data of other 23 meteorological stations in the territory of Latvia. Most of these stations also showed a decreasing tendency of the wind speed and at the same time no significant changes in the wind direction. The analysis of the changes in wind showed that it is very difficult to create homogeneous wind data series even for a comparatively short time periods. Changes in local environment at the vicinity of the meteorological stations (growing of trees and buildings) present the main reason of inhomogeneities in wind data series in the territory of Latvia However, a similar pattern of surface winds stilling in general has been found in the North Hemisphere [17]. This is consistent with the tendencies found in Europe, Australia, and USA. The studies that have analysed the wind speed data from terrestrial anemometers have generally found declines over the last 30–50 years [18–20], the cause of which is currently uncertain. The long-term changes of wind speed and direction in the territory of Latvia should be evaluated in the future taking into account the possible inhomogeneities. 

**Figure 5:**
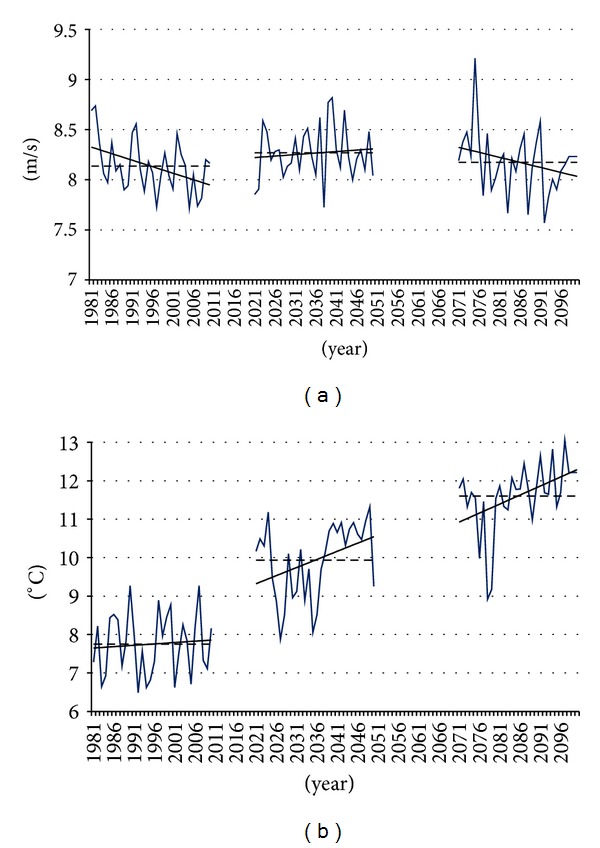
Time series of annual wind speed (a) and air temperature (b) for a point in the Baltic Sea (56.3937N, 20.6858E). Solid black lines show the tendencies for the respective time series. Dashed line reflects the 30-year average for the each period respectively.

The same decreasing tendency of wind speed over the sea for the period 1981–2010 has been shown by the CLM simulations. The historical trends of the wind speed were calculated for each grid cell in the study region. The results for one sample grid cell are presented in Figure 5. 

**Figure 6:**
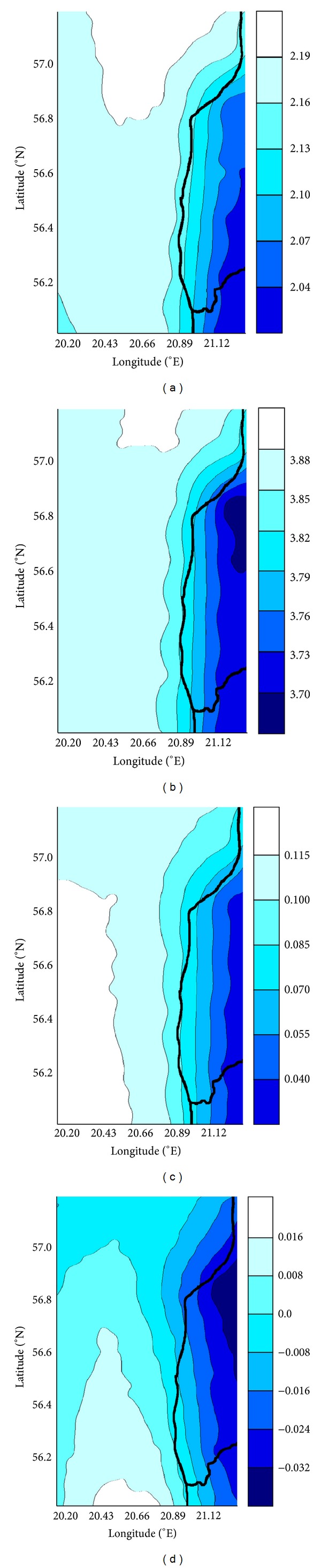
(a) Changes in the mean air temperature between 1981–2010 and 2021–2050. (b) Changes in the mean air temperature between 1981–2010 and 2071–2100. (c) Changes in the annual average wind speed between 1981–2010 and 2021–2050. (d) Changes in the annual average wind speed between 1981–2010 and 2071–2100.

Air density affects the energy density in the wind and hence the power output of wind turbines and is inversely proportional to air temperature, and thus increasing air temperature will lead to slight declines in air density and power production in the future. The effect is modest, but not negligible. It is found that at mean sea-level pressure an increase in air temperature by 5°C leads to a decrease in air density by 1-2% with a commensurate decline in energy density [18]. Downscaled results for the middle of the 21st century (2021–2050) exhibit a very significant increase in the air temperature from 2°C to 2.2°C in the covered territory (Figure 6(a)). However, very little differences (higher values) in the annual average wind speed were found in 2021–2050 relative to 1981–2010 (Figure 6(c)). Generally, the downscaled results for the end of the twenty first century (2071–2100) for a given regional climate model indicate higher magnitude changes of air temperature than those from the middle of the 21st century. The annual mean air temperature in the region will be from 3.7°C to 4.0°C higher than in 1981–2010 (Figure 6(b)). In 2071–2100 all grid points exhibit lower values of the mean wind speed relative to 2021–2050 and very little differences in the annual average wind speed relative to 1981–2010 (Figure 6(d)).

**Figure 7:**
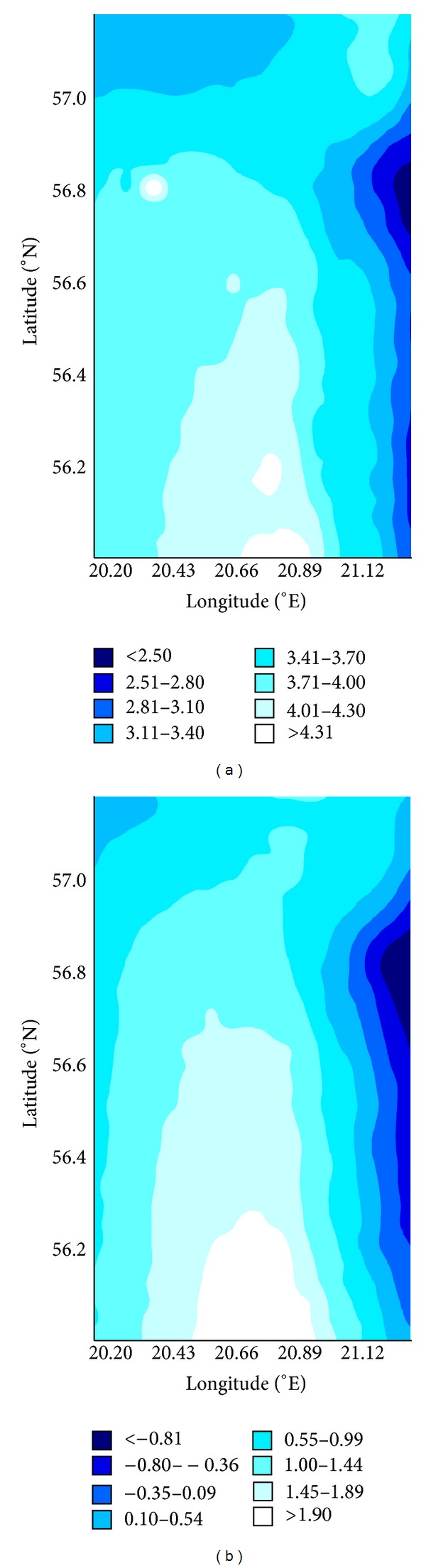
The percentage change in averaged annual wind power density for the periods 2021–2050 (a) and 2071–2100 (b) versus 1981–2010.

Figure 7 shows the projected percentage changes of 100 m annual mean wind speed for the periods 2021–2050 and 2071–2100 versus control period 1981–2010. 

Small changes (2–4%) were observed in the energy content of the wind for the middle of the 21st century. For the end of the 21st century the projections show an increase of the wind power density by 1-2% for offshore territories and neglected decrease (less than 1%) for coastal areas. 

It should be noted that the projections of the mean wind speed (and wind power) outlined in this paper should be viewed with caution since the climate change signal is of similar magnitude to the variability of the evaluation and control simulations. Also, the climate change projections are subject to a degree of uncertainty that limits their utility. Accordingly, future work should be focused on employing more models. 

## 4. Conclusions 

The regional climate model (CLM) and High Resolution Limited Area Model (Hirlam) simulations have been used to evaluate the wind power resources in the target territory. The results show that the model data are complementary to observations obtained from the meteorological stations and satellites and can therefore be used to usefully map long-term wind resources in large extensional offshore areas. The created maps provide a better estimate of the offshore wind resources than the previously available evaluation using the in situ observation data from the coastal stations. For offshore territories the wind power resources are predicted to be more than 500 W/m^2^. The results indicated that the offshore wind resources are promising for expanding national electricity generation and reducing the country's air emissions. A significant climate change signal—an increase in the air temperature—was found for the periods 2021–2051 and 2071–2100 relative to the climate reference period 1981–2010. Such shifts in the thermal regimes will likely be related to a decrease in the icing conditions and will result in less extreme cold conditions that will be the major advantage for installation, operation, and maintenance of wind turbines in the future. The recent studies [19, 20] emphasize the effects global warming may have on the harvesting of wind for energy production. According to the authors the wind energy resource may shrink in the future as climate warms in the large territories of Europe. The studies show the slight decrease of wind power output in most regions analyzed. In our study the different assessment method in smaller territory was used compared to these studies. However our data shows the same results: no significant changes in the average wind speed and interannual variability of annual and monthly wind speeds were found for the middle and end of the 21st century relative to 1981–2010. This means that wind energy resources will not change significantly during the 21st century. This work suggests that wind energy will continue to be a stable resource for electricity generation in the region over the 21st century. The future work should be focused on using more climate models as well as emission scenarios for assessing the possible future wind climate and wind power potential changes. 

## Figures and Tables

**Table 1:** Model simulations used.

Model (abbreviation)	Global model	Temporal resolution	Periods	Data
Regional climate model ETHZ-CLM_SCN_HadCM3Q0_25 km (CLM)	HadCM3	Daily (00:00 UTC)	1981–2010 2021–2050 2071–2100	Wind speed Wind direction Air temperature Atmospheric pressure

Atmospheric model DMI-Hirlam (Hirlam)	ECMWF	Hourly	2005–2010	Wind speed Wind direction Air temperature Atmospheric pressure

**Table 2:** In situ onshore weather stations used in the analysis.

Station	Distance from the closest coastline, km	Estimated roughness length	Meteorological parameter	Observation period	Temporal resolution
Liepaja 1	1.5	0.35	Wind speed Wind direction Air temperature Atmospheric pressure	1981–2010	3-hour (1981–2003) Hourly (2004–2010)

Liepaja 2	0.05	0.03	Wind speed Wind direction	2004–2010	Hourly

Pavilosta	0.3	0.74	Wind speed Wind direction Air temperature Atmospheric pressure	1981–2010	3-hour (1981–2003) Hourly (2004–2010)

**Table:** (a) MAD (m/s) between the simulated and observed long-term seasonal and annual wind speed (m/s) and air temperature (°C) data (1981–2010)

	Winter	Spring	Summer	Autumn	Year
Wind speed					
L1-CLM	−0.1	0.3	0.0	0.1	0.1
P-CLM	0.1	0.2	0.1	0.1	0.1
L2-CLM (2004–2010)	0.1	0.1	0.1	0.1	0.1
Air temperature					
L1-CLM	0.2	0.1	0.3	0.2	0.4
P-CLM	0.4	0.3	0.3	0.4	0.5

L1 is observation station Liepaja 1; L2 is observation station Liepaja 2; P is observation station Pavilosta. Data have been computed for the four standard climatological seasons (e.g., winter is identified by December, January, February, and so on).

**Table:** (b) Correlation coefficients between daily wind speed, air temperature, and atmospheric pressure (2004–2010)

Observation station-model	Winter	Spring	Summer	Autumn	Year
Wind speed					
L1-Hirlam	0.93	0.92	0.93	0.93	0.91
L2-Hirlam	0.84	0.90	0.92	0.93	0.86
P-Hirlam	0.86	0.91	0.92	0.92	0.89
Air temperature					
L1-Hirlam	0.98	0.98	0.95	0.98	0.95
P-Hirlam	0.97	0.99	0.95	0.98	0.93
Air pressure					
L1-Hirlam	0.99	0.99	0.99	0.99	0.99
P-Hirlam	0.98	0.99	0.98	0.99	0.99

L1 is observation station Liepaja 1; L2 is observation station Liepaja 2; P is observation station Pavilosta. Data have been computed for the four standard climatological seasons (e.g., winter is identified by December, January, February, and so on).

**Table 4:** Wind statistics at 100 m for 4 sites located in different distance from the sea coast (1981–2010).

Site	Coordinates			Wind speed, m/s	CV, %	Power density, W/m^2^
	Long	Lat	Dist, km	Annual Winter/summer	Annual Monthly	
I (198)	56.4834	21.0166	−1	7.9 8.6/7.0	3 9 (VI) 16 (IV, IX)	460
II (367)	56.9325	20.9391	7	8.8 10.0/7.6	3 10 (XII) 17 (IV, IX)	647
III (160)	56.3937	20.6858	15	8.9 10.2/7.6	3 10 (I, VI) 18 (IV)	671
IV (362)	56.9322	20.5285	26	9.1 10.3/7.9	3 10 (I, VI) 18 (IV)	711

CV: coefficient of variation is calculated as standard deviation/mean ∗ 100.

**Table 5:** Annual mean percentage of days with active wind speed (daily mean wind speed > 4 m/s).

Site	10 m	80 m	90 m	100 m
I	73	91	92	92
II	85	93	94	95
III	86	94	95	96
IV	89	94	95	96

The location characteristics of the sites are given in Table 4.
